# Steaming soil is effective in eliminating invasive alien plants (IAPs) – part II: effect of soil type

**DOI:** 10.1002/ps.8903

**Published:** 2025-05-15

**Authors:** Zahra Bitarafan, Wiktoria Kaczmarek‐Derda, Therese W Berge, Inger Sundheim Fløistad, Christian Andreasen

**Affiliations:** ^1^ Division of Biotechnology and Plant Health Norwegian Institute of Bioeconomy Research (NIBIO) Ås Norway; ^2^ Division of Forestry and Forest Resources Norwegian Institute of Bioeconomy Research (NIBIO) Ås Norway; ^3^ Department of Plant and Environmental Sciences University of Copenhagen Taastrup Denmark

**Keywords:** alien species, alternative weed control, invasive species, thermal soil disinfection, thermal weeding, weed seed control

## Abstract

**BACKGROUND:**

Soil disinfestation by steaming was evaluated due to its efficacy in controlling or potentially eradicating weed seeds. We exposed two soil types containing aggressive weeds to steam using a soil vacuum steaming method. The aim was to examine whether the method could be used to avoid the spreading of viable seeds to new regions when soil is reused.

**RESULTS:**

Dry seeds from two populations of *Avena fatua* and *Echinochloa crus‐galli* and one population of *Bromus sterilis*, *Lupinus polyphyllus*, and *Heracleum mantegazzianum* were incorporated in a medium sandy soil and a silty coarse sandy soil and examined for thermal sensitivity. Soil temperatures in the target range of 60–99 °C, followed by a 3‐min dwelling period, were tested. Increased soil temperature decreased seed germination. The two soil types did not influence the germination or viability response in most cases. For both populations of *A. fatua*, *B. sterilis,* and *E. crus‐galli,* a soil temperature of approximately 75 °C followed by a dwelling period of 180 s reduced the germination by about 90%. *Heracleum mantegazzianum* was more susceptible to heat than *L. polyphyllus* which required more than 100 °C to reduce seed germination by 90%.

**CONCLUSION:**

Soil steaming using a vacuum was an effective method to kill seeds of invasive alien plants (IAPs) in both soil types. However, the species showed different responses, indicating that steam temperature must be adapted to the specific weeds' susceptibility to heat. A temperature above 100 °C (or longer dwelling periods than 3 min) in the soil matrix might be necessary to kill all seeds. © 2025 The Author(s). *Pest Management Science* published by John Wiley & Sons Ltd on behalf of Society of Chemical Industry.

## Graphical abstract

Incorporation of propagative materials of selected invasive alien plant species in the soil. Steam treatment targeting different soil temperatures using a soil steaming prototype device. Fitted dose‐response curves showing the mortality rate obtained at different soil temperatures.

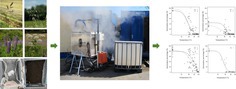

## 1 INTRODUCTION

Excavated construction and contaminated soils are frequently disposed of in landfills, and the recycling rate for high‐quality purposes is relatively low. Enhancing resource efficiency and reducing climate impacts is essential for sustainable soil management. The reuse of soil has the potential to minimize the environmental consequences linked to sourcing new soil.^1^ Nevertheless, the transportation and reuse of soils can introduce various invasive plants, diseases, and pests, leading to their spread. It is essential to eliminate their reproductive material (seeds, rhizomes, resting spores, etc.) before transferring the contaminated soil to new locations.^2^ Soil disinfestation involves substantially reducing pathogen and pest populations to a desired soil depth. This can be achieved by introducing a control agent that can penetrate the soil. Often, it is a volatile or liquid‐based substance applied or a heat treatment.^3^

Soil disinfestation with steam was first conceptualized as early as 1888 and gained widespread adoption during the 1960s. However, it was later replaced by more affordable chemical treatments. Nevertheless, certain chemical compounds, including methyl bromide, were phased out due to them being responsible for ozone depletion.^4^ The stringent regulatory pressure, particularly prevalent in Europe, prompted the search for alternative soil disinfection methods that are both effective and environmentally sustainable.^3^ The practice of soil disinfestation through steaming is currently being re‐evaluated in both open‐field and glasshouse horticulture due to its efficacy in controlling or potentially eradicating weeds, soil‐borne pathogens, and nematodes.^4^, ^5^ Nevertheless, this method comes with significant costs. It raises environmental concerns due to the labour intensive process and high consumption of fossil fuels.^6^ However, new technologies have been developed and utilized to expand their application and mitigate the costs and adverse effects, with significant cost reductions.^5^, ^7^ Various specialized machines have been designed specifically for soil disinfection using steam.^4^, ^8^, ^9^, ^10^, ^11^ The methods have been tested with positive results for agricultural and horticultural purposes.^12^, ^13^, ^14^, ^15^, ^16^

In this study, we evaluated the efficacy of a prototype stationary soil steaming device with a method resembling the method in commercial products (Soil Steam International AS, Stokke, Norway) in disinfecting soil masses. These experiments are an essential step towards evaluating the meaningfulness of scaling up the prototype to a potentially valuable tool for soil disinfection in soil relocation and reuse processes. Based on previous evidence of soil steaming's effectiveness against weeds in both glasshouse and field conditions, as well as preliminary research using the same steaming method^2^, ^6^, ^17^ we hypothesized that stationary soil steaming would make a meaningful contribution to disinfecting soils from weeds, facilitating a safer reuse of soil that would otherwise require disposal. The objective of this study was to determine the soil temperatures necessary to achieve varying degrees of control of selected weed species and test whether two different soil types (a medium sandy soil and silty coarse sandy soil) affected the estimated temperature requirements. Our study does not include the calculation of energy consumption and costs.

## 2 MATERIALS AND METHODS

In August 2022, a study was conducted in Ås, Norway, to assess the efficacy of steam treatment to eliminate seeds of selected weeds in two different soils.

### 2.1 Sample preparation before steaming

Norwegian seed samples of *Echinochloa crus‐galli* (L.) P. Beauv, *Avena fatua* L., *Bromus sterilis* L., *Lupinus polyphyllus* Lindl., and *Heracleum mantegazzianum* Sommier & Levier were selected. An *E. crus‐galli* and *A. fatua* seed sample from Poland were also included.

Mature seeds of the Norwegian populations were collected during September and October 2021 from Viken County and the Polish populations from Wadowice County. Seeds were collected from several individual plants. The pooled seeds were stored in paper bags under dry conditions at room temperature. Four replicates of 100 seeds of each species were counted and placed in polypropylene‐fleece bags (9 cm × 7 cm) except for *E. crus‐galli* seeds from Poland, *A. fatua*, and *B. sterilis* seeds, where 50 seeds per bag were used due to lack of seeds.

### 2.2 Steaming

To find the effective soil temperature to kill the species' seeds, we chose five target soil temperatures of 60, 70, 80, 90, and 99 °C, followed by a 3‐min dwelling period. Two commercial soil types were used provided by Lindum AS (Drammen, Norway). We used a medium sandy soil, and a silty coarse sandy soil. The results from the laboratory analysis of the soils (Eurofins Agro Testing Norway AS, Moss, Norway) are given in Table 1. Content of phosphorus (P), potassium (K), magnesium (Mg), calcium (Ca) and sodium (Na) were analysed using ammonium lactate and acetic acid with pH 3.75 for the extraction.^3^ Compared to the silty coarse sandy soil, the medium sandy soil had less clay, more organic matter, higher content of plant‐available P, a much higher content of plant‐available K, a slightly higher content of plant‐available Mg and Ca, and a slightly lower content of plant‐available Na. The moisture content of the medium sandy soil and the silty coarse sandy soil were 38.3% and 14.06%, respectively, before steaming.

**Table 1:** Characteristics of the two soil types in the study (results from commercial laboratory Eurofins Agro Testing Norway AS, Moss, Norway)

Characteristics		
Soil type	Medium sandy soil (2)	Silty coarse sandy soil (4)
Clay class	1 (<5%)	2 (5–10%)
Soil organic matter class	Organic matter rich soil (3)	Organic matter poor soil (1)
Organic matter content (% dry matter)	9.7%	1.7%
Loss on ignition (% dry matter)	9.7%	2.7%
pH	6.0	7.2
Phosphorus class	High (C2)	Low (A)
Potassium class	Very high (4)	Medium (2)
Phosphorus content, P‐AL (mg per 100 g air‐dried soil)	14	2
Potassium content, K‐AL (mg per 100 g air‐dried soil)	47	10
Magnesium content, Mg‐AL (mg per 100 g air‐dried soil)	19	12
Calcium content, Ca‐AL (mg per 100 g air‐dried soil)	200	180
Sodium content, Na‐AL (mg per 100 g air‐dried soil)	4	7
Volume weight (air‐dried soil)	1.0 kg L^−1^	1.3 kg L^−1^

*Note*: Phosphorus (P), potassium (K), magnesium (Mg), calcium (Ca) and sodium (Na) were analysed using ammonium lactate and acetic acid with pH 3.75.

**Figure 1:**
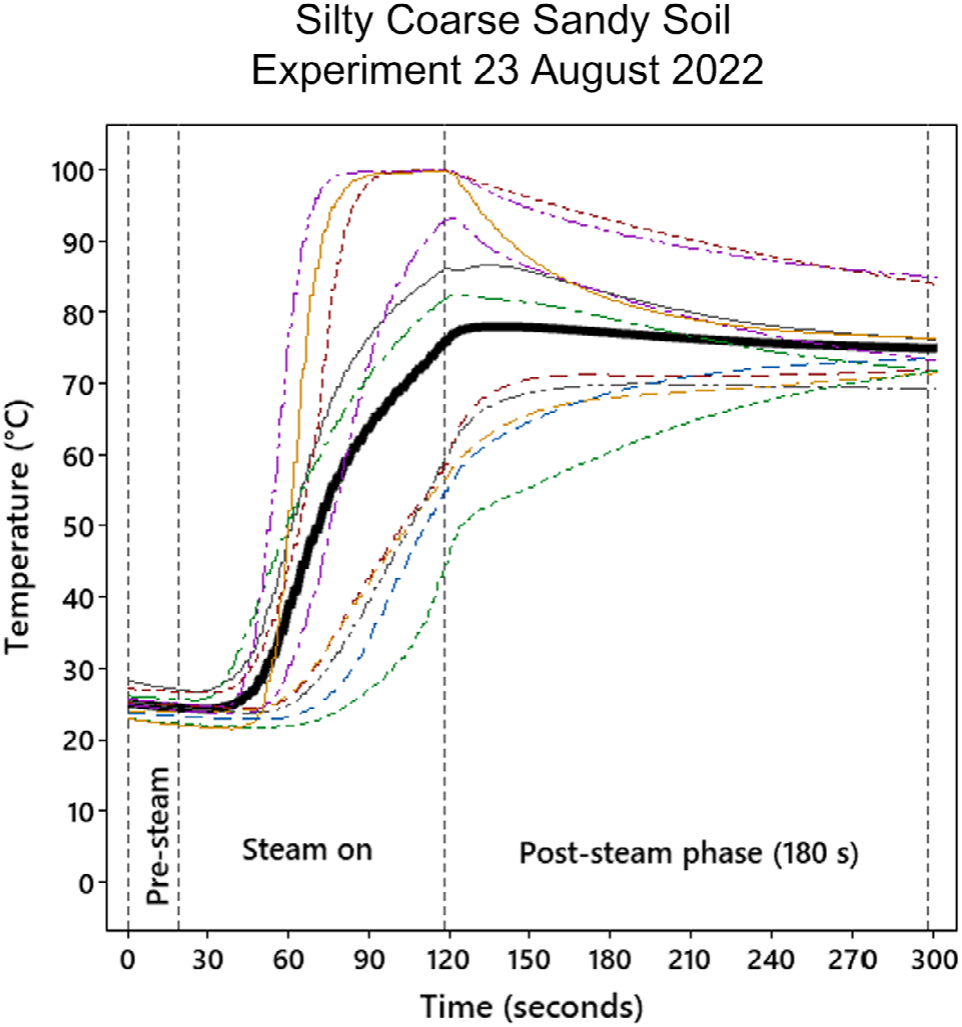
Example of soil temperature curves during steam treatment for target temperature 80 °C for one replicate basket (60 cm × 40 cm × 20 cm) with seeds of the five weed species and soil (soil volume approximately 60 cm × 40 cm × 7 cm). The mean soil temperature (black line) was calculated from the measured temperatures by 11 thermocouples (coloured lines). The maximum mean temperature was extracted and used to estimate parameters of the dose–response curves (cf. Fig. 2).

All seed bags in each replicate (combination of soil type and target temperature) were placed at the bottom of a plastic perforated basket container (60 cm × 40 cm × 20 cm) and covered with a 7 cm soil layer. In total, 40 baskets (5 target soil temperatures × 2 soil types × 4 replicates) were used. Each basket was placed in a steaming container (190 cm × 144 cm × 88 cm; effective steaming area of 120 cm × 80 cm), and 11 thermocouples were placed in different places in the soil. When the container lid was closed, steam was released from the top with a constant temperature of approximately 150 °C and vacuumed from the bottom. Soil temperature was recorded every second using PT1000 sensors connected to a cRIO‐9073 data logger (National Instruments Corp., Austin, TX, USA). A custom‐made program was used to record temperatures, the steaming and the post‐steaming duration (Fig. 1). Steaming and vacuum were stopped when the average temperature was about 5–10 °C below the target soil temperature because the temperature usually increased for some seconds after the steaming was stopped. The basket was removed from the steaming container when the post‐steaming duration of 3 min was completed. Samples were then removed immediately from the basket and warm soil into ambient air temperature (15–20 °C). The experiment was conducted two times. Hence, 80 baskets were exposed to steam (5 target soil temperatures × 2 soil types × 4 replicates × 2 experiments).

### 2.3 Germination and viability tests

After steaming, each bag was opened carefully, and the seeds were placed on the soil surface of soil‐filled plastic pots (*Ø* = 12 cm) and covered by a thin soil layer (2–3 mm). Commercial potting soil was used (Tjerbo Torvfabrikk AS, Rakkestad, Norway). It was composed of 80% sphagnum peat, 10% composted bark, 10% sand (*v/v/v*), limed, and fertilized with NPK (950:40:220 mg L^−1^) with a pH of 5.5–6.5. The pots were placed in a glasshouse (day/night: 21 °C/16 °C and 14 h/10 h; relative humidity: 68%) in a completely randomized design and watered from the bottom with tap water, and after that, as needed during the following 4 weeks where seed germination was recorded. Since *H*. *mantegazzianum* was a species of great interest, and its seeds germinated poorly in previous experiments, the seed viability was investigated with a tetrazolium test.^16^ However, this test was only used for seeds evaluated in the medium sandy soil. The tetrazolium test was performed by a commercial laboratory conducting seed analyses (Kimen såvarelaboratoriet AS, Ski, Norway).

### 2.4 Post‐processing of soil temperature data

The individual soil temperature measurement from each of the thermocouples was used to calculate the average soil temperature during steaming for each combination of target soil temperature and soil type in each replicate. The maximum values of each of the mean soil temperatures were extracted. An example of soil temperature curves is shown for the medium sandy soil (Experiment 1; temperature 80 °C; replicate 3) in Fig. 1. A comparison of the target and actual maximum mean soil temperature showed that achieving the precise target temperatures was difficult. The actual maximum mean soil temperatures in the eight replicates were 47.8–74.9, 60.5–84.2, 71.6–88.9, 84.01–95.5, and 88.4–99.3 °C for the target temperatures of 60, 70, 80, 90, and 99 °C. The length of the target post‐steaming duration of 180 s was generally obtained with five deviations. Actual periods were 177–193 s except for one case of 120 s. However, time did not have a significant effect on the seed germination.

### 2.5 Statistical analyses

For each soil type and species, the response to the soil temperature range was described by a three‐parameter log‐logistic dose–response curve model^18^:

(1) $$y=c+\left(d–c\right)/\left(1+\exp\;\left(b\left[\log\;\left(x\right)-\log\;\left({\mathrm{ED}}_{50}\right)\right]\right)\right),$$

where *y* is the response (germination percentage or viability percentage) 4 weeks after the treatment. The parameter *c* denotes the lower limit of the curve (0); *d* is a parameter close to the untreated control (upper limit). The parameter *b* is proportional to the slope of the curve at the dose *e*, which is the effective dose that reduces the response by 50% (ED_50_). The temperature of 25 °C was considered for untreated control samples.

The models were fitted using the extension package ‘drc’ for the software environment R.^19^, ^20^ The assessment of the individual fits was done by inspecting the graphical analysis of the residuals. *Post hoc* comparisons of parameters were based on pairwise *t*‐tests adjusted for multiple testing using the single‐step approach (Tukey's range test) implemented in the extension package drc multcomp.^21^

For each species, data from the two experiments were pooled because there was no significant effect from the experiments.

## 3 RESULT

**Figure 2:**
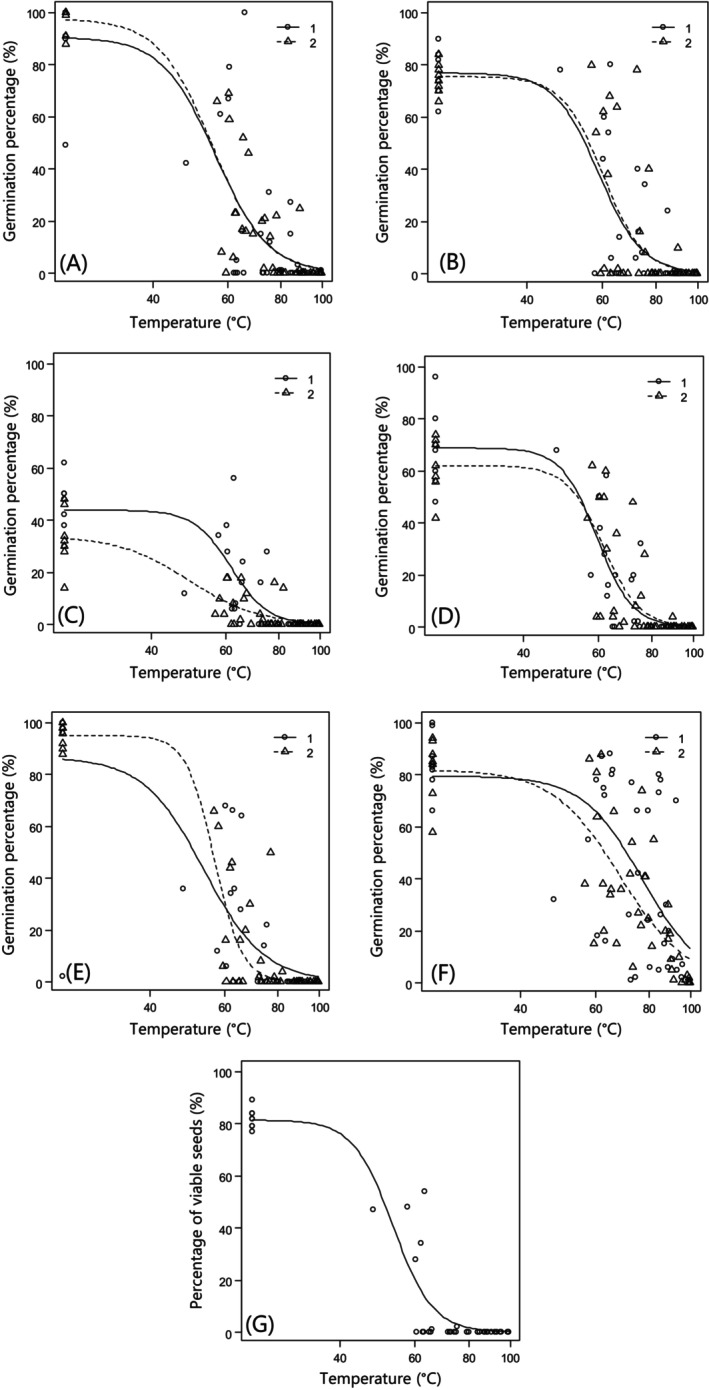
Seed germination (A–F) and viability (G) in response to increasing maximum soil temperatures during soil steaming in 1 (the medium sandy soil (straight lines estimated according to Eqn (1) (―); observation = ○)) and 2 (the silty coarse sandy soil (dashed line estimated according to Eqn (1) (− −); observation = ∆)), respectively. (A) *Echinochloa crus‐galli* from Norway, (B) *E. crus‐galli* from Poland, (C) *Avena fatua* from Norway, (D) *A. fatua* from Poland, (E) *Bromus sterilis* from Norway, (F) *Lupinus polyphyllus* from Norway and (G) *Heracleum mantegazzianum* from Norway.

Figure 1 shows an example of the temperatures measured by the 11 thermocouples during a soil steaming and post‐steaming period. The temperature varied substantially between the places where the thermocouples were mounted, and the mean temperature resulted from high and low temperatures. Increased soil temperature decreased seed germination (or seed viability) for all species (*P* = 0.001) in both soil types (Fig. 2). The soil type only affected the germination of the Norwegian *A. fatua* population significantly (*P* = 0.001). Higher temperatures were needed to reduce the seed germination in the medium sandy soil than in the silty coarse sandy soil (Table 2). However, there were no observations at temperatures between 25 °C and about 55 °C for the silty coarse sandy soil. That affects the precision of the estimation of the curve (Fig. 2), and, therefore, we suggest testing the steaming effect on *A. fatua* on the two soil types again.

**Table 2:** Effective dose (temperature) required to reduce the weed seed germination or (viability for *Heracleum mantegazzianum*) by 50% (ED_50_) and 90% (ED_90_) and the upper and lower 95% confidence limits of the estimates

Weed species (country)	Soil type	Effective dose (ED)	Estimate (± standard error)	Lower limit	Upper limit
*Echinochloa crus‐galli* (Norway)	1	50	56.08 (±3.6)	48.8	63.3
		90	76.8 (±4.6)	67.5	86.1
	2	50	55.1 (±3.1)	49.0	61.3
		90	75.7 (±4.4)	66.7	84.6
*Echinochloa crus‐galli* (Poland)	1	50	58.7 (2.6)	53.4	63.9
		90	75.8 (±5.0)	65.8	85.8
	2	50	60.1 (±2.1)	55.8	64.3
		90	76.2 (±5.3)	65.7	86.7
*Avena fatua* (Norway)	1	50	61.5 (±2.4)	56.6	66.5
		90	76.7 (±4.8)	67.1	86.2
	2	50	48.9 (±9.2)	30.5	67.4
		90	74.2 (±7.1)	60.2	88.3
*Avena fatua* (Poland)	1	50	59.4 (±1.8)	55.8	63.1
		90	73.2 (±3.6)	65.9	80.5
	2	50	61.4 (±1.7)	58.06	64.8
		90	76.2 (±4.0)	68.2	84.2
*Bromus sterilis* (Norway)	1	50	53.5 (±4.3)	44.8	62.2
		90	76.6 (±4.4)	67.7	85.5
	2	50	56.6 (±2.3)	51.9	61.3
		90	66.8 (±4.7)	57.3	76.3
*Lupinus polyphyllus* (Norway)	1	50	76.9 (±4.6)	67.6	86.3
		90	109.1 (±9.8)	89.6	128.6
	2	50	68.3 (±4.2)	59.9	76.7
		90	101.5 (±9.1)	83.3	119.7
*Heracleum mantegazzianum* (Norway)	1	50	53.2 (±2.3)	48.6	57.9
		90	67.3 (±2.0)	63.2	71.3

For *B. sterilis*, the germination curves for the silty coarse sandy soil had a lower relative slope (steepness of the curve) (Eqn (1)) than for the medium sandy soil (Table 3). In general, the slope of the curves did not differ much between the plant species. However, the germination percentages of untreated seeds (the controls) varied substantially between the plant species from almost 100% (*E. galli*) to less than 40% (*A. fatua*) (Table 2).

**Table 3:** Estimated parameters of the log‐logistic function (Eqn (1)) describing the relationship between seed germination/viability and maximum soil temperature caused by soil steaming

Weed species (country)	Soil type	Estimated parameter (± standard error)	
		*b*	*d*
*Echinochloa crus‐galli* (Norway)	1	6.9 (±2.2)**	90.4 (±6.3)***
	2	6.9 (±2.0)**	97.5 (±6.1)***
*Echinochloa crus‐galli* (Poland)	1	8.5 (±2.7)**	76.9 (6.0)***
	2	9.2 (±3.0)**	75.5 (±6.1)***
*Avena fatua* (Norway)	1	10.0 (±4.0)*	43.8 (±3.1)***
	2	5.2 (±2.9).	33.9 (±4.3)***
*Avena fatua* (Poland)	1	10.5 (±3.1)**	68.8 (±4.2)***
	2	10.1 (±2.7)***	62.1 (±4.3)***
*Bromus sterilis* (Norway)	1	6.1 (±1.7)***	86.6 (±6.5)***
	2	13.2 (±8.3)	95.1 (±5.8)***
*Lupinus polyphyllus* (Norway)	1	6.2 (±2.1)**	79.5 (±8.4)***
	2	5.5 (±1.4)***	81.9 (±8.2)***
*Heracleum mantegazzianum* (Norway)	1	9.4 (±2.1)***	81.4 (±3.4)***
	2	—	—

Significance codes: 0 ‘***’ 0.001 ‘**’ 0.01 ‘*’ 0.05 ‘.’ 0.1 ‘’ 1.

*Note*: Parameter *b* denotes the relative slope around effective dose that reduces the response by 50% (ED_50_), and parameter *d* denotes the upper limit (the maximum recorded seed germination).

Species responded differently to the soil temperature (Fig. 2, Tables 2 and 3). For both populations of the species *A. fatua* and *E. crus‐galli*, a soil temperature of approximately 75 °C (followed by a dwelling period of 180 s) was enough to reduce seed germination by 90% (Table 2). This was also achieved for *B. sterilis* in the medium sandy soil (Table 2). Among the two non‐agricultural weed species, *H. mantegazzianum* was most susceptible to heat, while *L. polyphyllus* was the most resistant species, and it required soil temperatures of more than 100 °C to reduce seed germination by 90% (Table 3). In general, the 95% confidence intervals for the estimated effective dose (ED) values were wide (Table 2).

## 4 DISCUSSION

When soil is subjected to a steam treatment, it disrupts its natural thermal equilibrium, inducing a multiphase high‐temperature flow through its pores and rapidly elevating its temperature.^4^ Consequently, the elevated temperatures achieved through steam treatment effectively result in seed thermal death. The success of soil steaming relies on three key factors: the temperature, the duration of exposure to the hot soil, and the soil depth exposed during the treatment.^22^ Soil steaming of the ground can, under optimal soil conditions, effectively disinfest clay soils *in situ*. Lethal soil temperatures are often only attained in the upper layers of sandy and loam soils. Steaming of peat soils is not particularly effective due to their high water retaining capacity.^23^ However, by creating negative pressure with vacuum steaming, the time for achieving a target temperature might vary between different soil types, but the target depth will be reached. It has also been demonstrated that vacuum steaming, creating a negative pressure, is more efficient in disinfecting sand, loam, clay, and peat soils.^23^

Excavated soils are usually not homogeneous masses but may consist of many different materials, for example, stones of various sizes, twigs, roots and partly decomposed organic material, different sizes of hard or loose clods of earth (where seeds can be more or less protected from soil steaming), and mixtures of varying soil types. Therefore, we expect a significant variation in the germination and viability response to the same soil steaming temperature because the steam may heat some parts of the soil faster than others. Figure 1 shows an example of the soil temperatures measured in one experimental basket containing a soil mass of approximately 60 cm × 40 cm × 7 cm. The thermocouples measured the temperature at different places in this soil mass and showed that the temperature course varied significantly within the basket. The mean temperature was an average of relatively high and low temperatures. A significant variation in the germination and viability data can also be observed in Fig. 2. A soil temperature of about 90–100 °C seems necessary to avoid a large variation in the germination and viability response and ensure disinfected soil that can be moved to other places without any risks of spreading the tested viable weed seeds.

All seeds were dry when placed in the soil steamer, but soil and seed moisture affect the seeds' susceptibility to heating.^24^, ^25^ Soil moisture near field capacity has yielded high heating efficacy of steaming disinfestation methods.^4^ Dry seeds heat up slower than imbibed seeds because water is a better heat conductor than dry tissue. Seeds in the soil seed bank may be imbibed, and therefore, we expect a lower temperature would be sufficient to kill imbibed seeds than dry seeds. Enzymes in imbibed seeds are sensitive to high temperatures. When the temperature approaches 40–45 °C, large proteins often start breaking down or changing structure, resulting in poor enzyme activity and failure of normal germination.^25^, ^26^ The optimum germination temperature for most seeds is between 15 and 30 °C. The maximum temperature is between 30 and 40 °C.^27^ Some dry seeds are better protected against hot steam than others, for example, if they have a thick seed coat or are protected by other tissue. For example, the ability of *E. crus‐galli* seeds to withstand high temperatures has been ascribed to its seed structure, protection of the caryopsis by its lemma and palea, sterile floret, and the first and second glume.^28^

Many factors during soil heating are essential to reduce seed germination: maximum heat duration,^29^ temperature attained,^12^, ^16^, ^30^ seed water content, soil moisture,^31^ seed structure, anatomy and morphology (e.g., size, seed coat),^32^ as well as seed dormancy.^16^ However, heat duration and maximum temperature are the most critical factors for reducing seed germination. Dahlquist *et al*. showed that the duration of exposure to kill seeds varied from 0.17 h at 70 °C to 672 h at 39 °C.^34^

We measured the seed viability of *H. mantegazzianum* using the tetrazolium test because of seed dormancy. Moravcová *et al*.^35^ found that *H. mantegazzianum* seed dormancy was not completely broken until the first spring but that some seeds re‐enter or retain dormancy during high summer temperatures and that the threshold needed for breaking the dormancy was achieved gradually during the cold autumn and winter months. However, the dormancy‐breaking process took several years in a small fraction of seeds.^33^ Still, viable seeds may not be able to germinate.

## 5 CONCLUSION

The two soil types were quite similar, with a high sand content. Soil steaming had the same effect on most of the seeds in both soil types with different moisture content. Testing other soil types (e.g., clay soil and peat soil) may have resulted in a significant soil effect due to different soil water‐holding capacities and porosity. It is not obvious why soil type significantly affected seed germination of the Norwegian *A. fatua* seeds.

*Lupinus polyphyllus* seeds were the most resistant to steaming, with an average ED_90_ value (across soil types) of approximately 105 °C (Table 3). However, it is not possible to rank the four other species due to the small differences and the large variation in the data, but all had mean ED_90_ values between 67 and 77 °C. A temperature of above 100 °C (or longer dwelling period than 3 min) in the soil matrix might be necessary to kill all seeds if they are dry. Seeds in the soils may be imbibed at the time of steam treatment, and therefore, a lower temperature may be sufficient.

## CONFLICT OF INTEREST STATEMENT

No conflict of interest has been declared by the authors. The funders had no role in the design of the research, data collection and analysis, writing of the manuscript, or the decision to publish the results.

## AUTHOR CONTRIBUTIONS

Zahra Bitarafan: experimental design, implementation of the study and data collection, data analysis and interpretation of the results, writing – original draft, reviewing and editing. Wiktoria Kaczmarek‐Derda: conceptualization, funding acquisition, implementation of the study. Therese W. Berge: implementation of the study – responsible for the temperature data. Inger Sundheim Fløistad: conceptualization, funding acquisition, supervision. Christian Andreasen: supervision, writing – original draft, reviewing and editing. All authors reviewed and approved the final version of the manuscript.

## Data Availability

The data that support the findings of this study are available from the corresponding author upon reasonable request.
